# Synchrotron X-ray fluorescence microscopy-enabled elemental mapping illuminates the ‘battle for nutrients’ between plant and pathogen

**DOI:** 10.1093/jxb/erab005

**Published:** 2021-01-13

**Authors:** Fatima Naim, Karina Khambatta, Lilian M V P Sanglard, Georgina Sauzier, Juliane Reinhardt, David J Paterson, Ayalsew Zerihun, Mark J Hackett, Mark R Gibberd

**Affiliations:** 1 Centre for Crop and Disease Management, School of Molecular and Life Sciences, Curtin University, Bentley, Western Australia, Australia; 2 School of Molecular and Life Sciences, Curtin University, Bentley, Western Australia, Australia; 3 Australian Synchrotron, ANSTO, Clayton, Victoria, Australia; 4 Hasselt University, Belgium

**Keywords:** Asymptomatic tissue, crop fungal disease, nutrient mapping, nutrient re-distribution, plant–pathogen interaction, *Pyrenophora tritici-repentis*, synchrotron, wheat, X-ray fluorescence microscopy, yellow spot

## Abstract

Metal homeostasis is integral to normal plant growth and development. During plant–pathogen interactions, the host and pathogen compete for the same nutrients, potentially impacting nutritional homeostasis. Our knowledge of outcome of the interaction in terms of metal homeostasis is still limited. Here, we employed the X-ray fluorescence microscopy (XFM) beamline at the Australian Synchrotron to visualize and analyse the fate of nutrients in wheat leaves infected with *Pyrenophora tritici-repentis*, a necrotrophic fungal pathogen. We sought to (i) evaluate the utility of XFM for sub-micron mapping of essential mineral nutrients and (ii) examine the spatiotemporal impact of a pathogen on nutrient distribution in leaves. XFM maps of K, Ca, Fe, Cu, Mn, and Zn revealed substantial hyperaccumulation within, and depletion around, the infected region relative to uninfected control samples. Fungal mycelia were visualized as thread-like structures in the Cu and Zn maps. The hyperaccumulation of Mn in the lesion and localized depletion in asymptomatic tissue surrounding the lesion was unexpected. Similarly, Ca accumulated at the periphery of the symptomatic region and as microaccumulations aligning with fungal mycelia. Collectively, our results highlight that XFM imaging provides the capability for high-resolution mapping of elements to probe nutrient distribution in hydrated diseased leaves *in situ*.

## Teaser

Synchrotron X-ray fluorescence microscopy elemental maps highlight major nutrient re-distribution during disease progression.

## Introduction

Plants rely on finely balanced transport and distribution of mineral nutrients to support normal growth and development. Deprivation of macronutrients such as potassium (K) and calcium (Ca) results in major developmental defects and yield losses (Loneragan and Snowball, 1969; Pettigrew, 2008). Micronutrients such as copper (Cu), iron (Fe), manganese (Mn), and zinc (Zn) are also essential in smaller quantities to support structural integrity and as cofactors of enzymes involved in detoxification of products generated during oxidative stress (Hall and Williams, 2003). During plant–pathogen interactions, the normally tightly controlled metal homeostasis is challenged as both compete for the same nutrients; that is, nutrients essential for plant growth and development such as metals are also important in fungal virulence, growth, and disease development (Gerwien *et al.*, 2018).

Plant pathogens have evolved to invade host plant tissue to acquire nutrients. Pathogenic fungi, for example, employ various modes of host cell manipulation, which enables the fungi to extract nutrients from the host plant tissue, proliferate, complete their life cycle, and spread and colonize other healthy tissues (Fernandez *et al.*, 2014). Specific examples are necrotrophic fungi, which elicit toxins to initiate plant cell death, enabling the fungi to extract nutrients from the dead tissue. These toxins may be cell wall-degrading enzymes, proteins, or secondary metabolites that promote necrotization of plant tissue and proliferation of the fungal pathogen (Faris and Friesen, 2020).

Plants, on the other hand, are capable of detecting pathogen invasion and early responses by the plant against the pathogens including: re-distribution of intracellular calcium, activation of the hypersensitive response (HR) pathway, reactive oxygen species (ROS) bursts, the production of antimicrobial compounds, and cell death (Alvarez *et al.*, 1998; Torres *et al.*, 2006; Bose *et al.*, 2011; Aldon *et al.*, 2018). Host defence mechanisms against necrotrophic fungi have been shown to be complicated due to the complexity of the mode of attack deployed by various necrotrophs (Thomma *et al.*, 2011; Williams *et al.*, 2011). Host plants can also employ defence strategies against pathogens that involve regulation of nutrients: one strategy is to sequester essential nutrients away from the pathogen to hinder pathogen growth (Hood and Skaar, 2012; Núñez *et al.*, 2018; Cabot *et al.*, 2019) or, secondly, the host may produce localized nutrient accumulation, at levels toxic to the pathogen (Poschenrieder *et al.*, 2006). These processes through which the host regulates nutrient concentration and distribution to fight the pathogen are referred to as nutritional immunity (Gerwien *et al.*, 2018).

Despite the concept of nutritional immunity being well established, visualization of the spatial and temporal changes to nutrient homeostasis that occur within plant tissues infected with a pathogen historically has been technically challenging due to the lack of tools capable of directly imaging nutrients at the cellular level in plant tissue. Such a capability is now provided by X-ray fluorescence microscopy (XFM) (Kopittke *et al.*, 2018; van der Ent *et al.*, 2018; Rodrigues Marques *et al.*, 2020) which offers exciting opportunities to further explore the mechanisms of nutritional immunity directly associated with plant resistance and/or tolerance to diseases.

Synchrotron-based XFM provides direct, simultaneous, multielement mapping, and has been an important analytical tool to study metal homeostasis in plants since the late 1990s (Punshon *et al.*, 2009). However, the recent advances in synchrotron-based XFM technology, such as improved brightness of third-generation synchrotron X-ray sources and improved detection and data collection speeds, make it possible to simultaneously capture elemental mapping at cellular or near cellular spatial resolution *in situ* or *in vivo* (Kopittke *et al.*, 2018; van der Ent *et al.*, 2018). The maps are generated in experimentally realistic time frames, which are required for detailed biological studies. In recent years, the technique has been used to show compartmentalization of various metals and their accumulation in a diverse range of plant tissues (such as leaves, roots, and grain), as well as to visualize biofortification of important micronutrients in genetically modified crop plants (Wang *et al.*, 2013; Cheah *et al.*, 2018; van der Ent *et al.*, 2018; Beasley *et al.*, 2019).

Yellow (tan) spot is a devastating foliar disease of wheat caused by the necrotrophic fungus *Pyrenophora tritici-repentis* (Died.) Drechs. Yellow spot appears as tan-coloured necrotic lesions often surrounded by chlorotic tissue in wheat leaves (Lamari and Bernier, 1989, 1991). The frequency and size of necrotic and chlorotic tissues effectively reduce the size of the photosynthetically active leaf area, resulting in wheat yield losses of up to 50% (Faris *et al.*, 2013). The fungus secretes a combination of host selective toxins (effectors) which upon entry interact with host genes, resulting in inhibition of photosynthesis, accumulation of ROS, and cell death (Manning *et al.*, 2009; Pandelova *et al.*, 2012). Although yellow spot is a prominent disease found in major wheat-growing regions of the world, past studies have largely focused on effector-mediated host–pathogen interactions (Pandelova *et al.*, 2012; Moffat *et al.*, 2014; Moolhuijzen *et al.*, 2018), and there is limited knowledge on other aspects of interaction between the host and pathogen that may contribute to our capacity to select for host resistance and tolerance.

Here we report the use of synchrotron-based XFM technology to map partitioning of essential macro- and micronutrients (K, Ca, Mn, Fe, Cu, and Zn) in wheat leaves infected with *Pyrenophora tritici-repentis* (Ptr). Using XFM, we have monitored nutrient distribution during plant–pathogen interactions over a time course of 8 d. The XFM-generated elemental maps showed a clear re-distribution of leaf K, Ca, Mn, Fe, Cu, and Zn in and around the necrotic lesion in infected leaves, providing new insights and possibilities to probe plant–pathogen interactions along an axis of nutrient acquisition and re-distribution.

## Materials and methods

### Plant and fungal material and inoculation

A spring wheat (*Triticum aestivum* cv. Scout) was used for the study. This cultivar is rated as susceptible–very susceptible for yellow spot disease caused by Ptr (See *et al.*, 2018). Untreated Scout seeds were sown in potting soil (UWA custom mix, Richgro, Jandakot, Australia) in 4.5 litre pots. Plants were grown and maintained in a glasshouse on Curtin University Bentley Campus during the spring months (October to November). Each pot contained one plant, and pots were fertilized (~10 g of Nitrophoska^®^) 4 weeks after sowing the seeds. The glasshouse temperature ranged between 18 °C and 28 °C throughout the experiment.

The Ptr race 1 isolate M4 (known to produce the effectors ToxA and ToxC) was used in this study (Moffat *et al.*, 2014). This isolate collected in 2009 from Meckering, Western Australia was cultured as previously described (Moffat *et al.*, 2014). Conidia were harvested by flooding the plates with ultrapure water and gently detaching the conidia using an L-shaped plastic spreader. Although the harvest technique results in a mixture of conidia and fungal mycelial fragments, for inoculation of leaves, the concentration of conidia was adjusted to ~3000 conidia ml^–1^ in ultrapure water and a 10 µl droplet was placed on the youngest fully unfolded wheat leaf when plants were 5 weeks old (Zadoks stage 24–25). To ensure the droplet stayed on the leaf, wheat leaves were gently attached to a flat platform with double-sided tape prior to inoculation and remained situated there throughout the duration of the experiment. For 48 h following inoculation, to facilitate infection, the glasshouse relative humidity was maintained at ≥95% using a fitted misting system (Idrobase^®^ Fog Extra, Italy). The control uninoculated plants and inoculated plants were maintained in the same environment throughout the duration of the experiment.

The experiment was set out as a completely randomized design at the plant level. Two treatments, control (mock inoculated with water) and inoculation with Ptr conidial suspension, were applied to the same plant as a biological replicate to reduce the influence of individual plants on the treatment interaction. On each plant, the two youngest fully unfolded leaves were used for each treatment. Each leaf contained two treated regions (a total of four regions analysed by XFM for each treatment at each time point). Leaves were sampled at 2-, 4-, and 8-days post-inoculation (dpi). Leaves were bagged individually in zip lock bags which were then placed in a plastic container, stored at 4 °C, and transported on ice to the Australian Synchrotron (Melbourne, Australia). The Ptr development pauses at lower temperatures (da Luz and Bergstrom, 1986) and therefore leaves were stored at 4 °C and imaged within 3–5 d post-harvest. Wheat leaves are relatively hardy and remained fresh during the transportation. Samples were analysed for fungal staining and XFM analysis at room temperature.

### Staining of fungal mycelium within the leaf

Fungal mycelia in infected wheat leaves were stained as previously described (Williams, 2013). Essentially, ~1.5 cm sections of infected and control leaves were fixed and cleared overnight in a 1.5 ml solution of acetic acid:ethanol:chloroform (1:6:3). The leaf sections were then transferred to 1 ml of staining solution (trypan blue:lactophenol, 1:1) and heated in a boiling water bath for 5 min followed by destaining in 1 ml of ethanol:lactophenol (2:1) for 10 min. The stained sections were placed on a slide with two drops of 60% glycerol and imaged using a microscope (Olympus BX51, Olympus Corp, Japan). A total of three biological replicates for each time point and treatment were analysed.

### X-ray elemental mapping

To examine the *in situ* distribution of a range of elements in infected wheat leaves, we used the XFM beamline at the Australian Synchrotron (Paterson *et al.*, 2011; Howard *et al.*, 2020) Leaf samples from the infected and control treatments were mounted onto the single aperture Perspex mount with 100 mm×100 mm aperture area using double-sided tape (Supplementary Fig. S1). Initially, the incident monochromatic beam (18.5 keV) with a spot size of 2 μm (~2.35 σ) was focused, using a KB mirror pair, onto the sample, and the X-ray fluorescence emitted by the sample was collected in a back-scatter geometry with the 384-element Maia (rev D) detector as previously described (Kirkham *et al.*, 2010; Howard *et al.*, 2020). Subsequently, to increase sample throughput, a 10 μm pixel size was used. Therefore, high-resolution (2 μm) elemental maps were generated for one representative sample for each time point and treatment, and the rest were imaged at the lower resolution (10 μm). An ~1 cm^2^ region of the infected leaf was scanned which included the inoculated region; the scan time was 0.5 ms per pixel, with per pixel counts maintained at <10 000 counts s^–1^, per detector pixel-element (Supplementary Fig. S2). A similar area and region of the equivalent control leaf was also scanned. The high- and low-resolution scans took ~120 min and 20 min per scan, respectively. The data collected from the Maia detector were analysed using CSIRO’s GeoPIXE® software to generate true-elemental images (Ryan, 2000). Fluorescence emission data were converted into quantitative elemental maps through calibration against Mn, Fe, Pt, Y, Cu, and Ti elemental foils (Micromatter, Canada) with known composition and density, as previously described in the literature (Ryan, 2000). The average of the six calibration foils measured was used in this analysis (SD of 6.7%). The quantitative elemental maps were exported from GeoPIXE as tiff files, which were then visualized and concentrations determined within regions of interest using ImageJ (Schindelin *et al.*, 2015). The sample attenuation of X-ray emission is element dependent, with lower energy X-rays from lighter elements being attenuated to a greater extent than the higher energy X-ray emission from heavier elements, and this is an important consideration with data interpretation, as highlighted by others (Szczerbowska-Boruchowska, 2012). The transmission of X-ray energies corresponding to Kα emission lines of P, S, Cl, K, Ca, Mn, Fe, Cu, Zn, As, Se, Br, and Rb, for various thicknesses of a hydrated sample approximated to water are shown in Supplementary Fig. S3 (Henke *et al.*, 1993). The plots highlight that attenuation is substantial for elements lighter than Ca, for a 200 µm thick sample (>85% attenuation of signal). However, attenuation is <55% for elements heavier than Mn. Therefore, elemental maps for P, S, Cl, K, and Ca will be heavily influenced by elemental distribution at the surface of the sample, and therefore should be considered as semi-quantitative, consistent with the literature (Szczerbowska-Boruchowska, 2012), which we have taken into consideration when interpreting our findings. We show, in Supplementary Fig. S4, a comparison of areal density maps and the areal density maps normalized to scattering. Similar patterns of elemental enrichment are seen across both sets of images, providing confidence that the regional differences in element content observed in this study are not due to sample thickness effects. The limits of detection, calculated as 3 σ of the signal measured from an experimental blank (6 µm thick mylar) were 35, 27, 21, and 18 ng cm^–2^ for Mn, Fe, Cu, and Zn, respectively.

X-ray photodamage is an important consideration for all X-ray experiments, and is influenced not only by the radiation dose, but also by the atmosphere in which the sample is analysed (e.g. the presence or absence of oxygen) and the form of the sample (e.g. hydrated and analysed at room temperature, hydrated and analysed at cryogenic temperature, or dehydrated), as reported by multiple authors (George *et al.*, 2012; Hackett *et al.*, 2012; Jones *et al.*, 2017; Xiao *et al.*, 2017). Based on the study of Jones *et al*. (2017), we estimate the radiation dose as 54 Gy for the longest dwell time of 0.2 ms per (2 µm×2 µm) pixel used in this study. This is four orders of magnitude smaller than the minimum dose at which elemental re-distributions occurred in the study by George *et al.* (2012), Hackett *et al.* (2012), and Jones *et al.* (2017). In our study, no visible effects of photodamage such as distortion of leaf anatomy or discoloration of imaged leaf surfaces were observed. However, changes in elemental oxidation state or chemical form that may have occurred during measurements cannot be ruled out, and these changes may have been evident at a spatial scale below the spatial resolution that was used in this study. Even if such changes were to occur, they would not have a substantive impact on the interpretation of results presented here due to relative effects likely to be similar for all treatments, and, more importantly, we have analysed total nutrient content and not speciation (oxidation state) of elements.

### Data analysis

Following determination of the spatial distribution of elements, ANOVA was used to evaluate effects of treatments (control versus inoculated) and duration of infection (2, 4, and 8 dpi) on the concentration of elements in the imaged tissue. The data were analysed for four replicates for each time point and treatment (control and diseased). Two of these replicates were on the same leaf, with one treatment closer to the base of the leaf and the other closer to the tip of the leaf. Therefore, for each time point, two leaves with two treatment points were used to generate four control and four diseased samples. The only exception was that only three replicates were analysed for infected treatments at 2 dpi. Additionally, we examined whether the concentration of elements varied up- and downstream of the symptomatic zone. Data visualization was performed in RStudio using the ggplot2 package.

## Results

### XFM enables visualization of the distribution of key nutrients in fungi-infected wheat laminae relative to anatomy and location of fungal mycelia

**Fig. 1:**
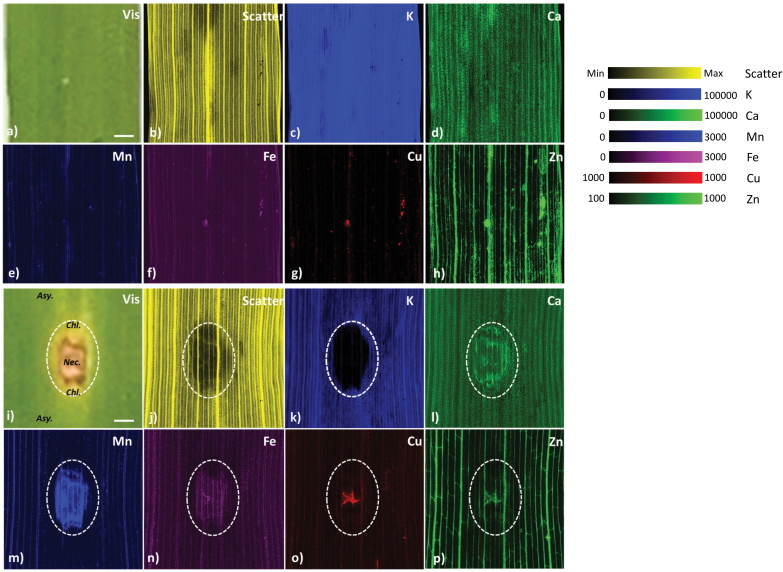
. XFM-generated elemental maps of a control wheat leaf (A–H) and an age-matched Ptr-infected wheat leaf (I–P), harvested 8 days post-inoculation (dpi). (A, I) Light image of the region of the leaf scanned, (B, J) Compton scatter map of leaf density, (C, K) K, (D, L) Ca, (E, M) Mn, (F, N) Fe, (G, O) Cu, and (H, P) Zn elemental maps. The necrotic (Nec.) and chlorotic (Chl.) zones forming the symptomatic zone are indicated by a dashed white line with the asymptomatic (Asy.) zone defined outside this region (A). Each map is false coloured to help visualize the patterns of elemental distribution, with colour maps provided. To best highlight the Cu distribution in mycelia, a binary colour scale was used (i.e. 1000–1000). Scale bar=1 mm, and elemental maps are expressed in units of ng cm^–2^.

XFM mapping was used to reveal elemental distributions of K, Ca, Mn, Fe, Cu, and Zn in relation to key features of leaf anatomy, which enabled assessment of alterations to leaf nutrients during the process of fungal disease development. Representative optical images in addition to XFM false-colour Compton scattering and elemental maps (K, Ca, Mn, Fe, Cu, and Zn) from a control wheat leaf sample (Fig. 1A–H) and an age-matched infected sample (8 dpi with Ptr) (Fig. 1I–P) were compared. The optical image of a Ptr-infected sample (Fig. 1I) shows the damage caused by the necrotrophic fungus, as a characteristically distinct central necrotic lesion surrounded by a chlorotic zone. The chlorotic zone extends beyond the necrotized tissue and fades away into asymptomatic tissue, apical and basal to the lesion (outlined on Fig. 1I). Hereafter, collectively, we refer to the necrotic and chlorotic zones as the symptomatic region. No signs of Ptr infection were observed in the optical images of control leaves, which displayed normal leaf morphology (Fig. 1A). Compton scatter images (Fig. 1B, J), which indicate tissue density, could be used to confirm the location of anatomical structures such as venation, in laminae from both control (Fig. 1B) and infected treatments (Fig. 1J). Whereas the vascular tissues were enriched in all elements studied, a specific Zn enrichment was also observed within the trans-veins running generally orthogonal to, and interconnecting, the main veins (Fig. 1H, P). In addition to revealing leaf anatomy, the Compton scattering maps of infected tissues (not unexpectedly) showed a drastic loss of tissue density within the necrotic and chlorotic zones (Fig. 1J). Compton scattering maps and optical images were therefore used to define the location and extent of symptomatic regions, and thus the boundary between symptomatic and asymptomatic regions of laminae in XFM data, which allowed comparative analysis of nutrient concentrations in laminae with respect to symptomatic and asymptomatic regions from either the infected or control leaf samples.

The distribution maps of all elements (K, Ca, Mn, Fe, Cu, and Zn) showed changes within the symptomatic tissue by 8 dpi (Fig. 1K–P). Specifically, the comparative analysis of control and infected samples showed that: K became depleted within the symptomatic tissue, as visualized by the reduced intensity in the false-colour image (Fig. 1C versus Fig. 1K); Ca showed a complex distribution of decreased concentration within the necrotic core, but an increased concentration at the periphery of the symptomatic zone (Fig. 1D versus Fig. 1L); Mn showed pronounced accumulation throughout the symptomatic region and its distribution pattern was highly disrupted in vascular tissue apical and basal of the symptomatic region (Fig. 1E versus Fig. 1M); Fe showed overall low levels of accumulation in the symptomatic region, with highly localized accumulation in a ‘thread-like’ structure at the centre of the necrotic core (Fig. 1F versus Fig. 1N); and Cu and Zn accumulated within the‘thread-like’ structures, that appeared like fungal mycelia (Fig. 1G versus Fig. 1O for Cu and Fig. 1H versus Fig. 1P for Zn). As Cu concentrations were low throughout the tissue, the patterns of intense Cu accumulation could be used to identify the localization of the fungal mycelia in the infected tissue. Closer inspection of the fungal mycelia deposits revealed that Fe only co-localized in a small portion of mycelia compared with co-localization of Cu and Zn (Fig. 1N versus 1O and 1P).

### XFM elemental maps reveal altered nutrient distribution over an 8 d time-course of wheat leaves infected with *P. tritici-repentis*

**Fig. 2.**
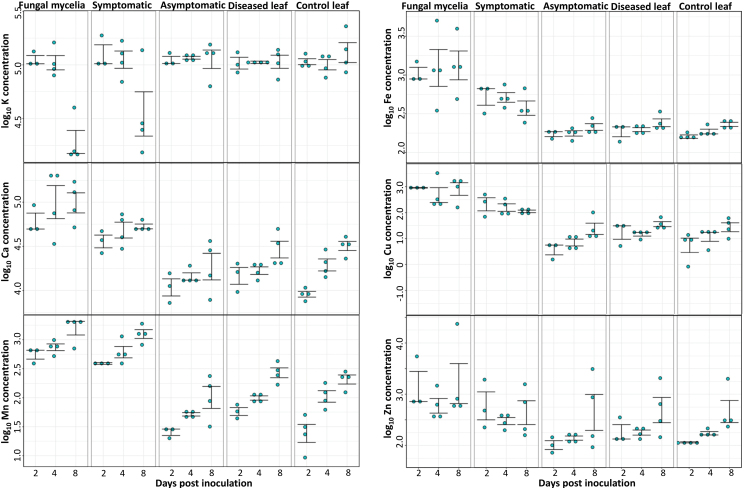
Scatter plots of elemental concentrations log_10_ transformed for K, Ca, Mn, Fe, Cu, and Zn for various regions of interest (ROI) within infected sites and in surrounding leaf tissue, in Ptr-infected wheat leaf sections harvested at 2, 4, and 8 dpi. Plots were jittered along the *x*-axis and centred on the sampling dates for better visualization. Elemental concentrations are calculated for ROIs in the infected leaf sample: fungal mycelia, symptomatic, asymptomatic depicting where the fungal body was present based on Cu accumulation, visible lesion based on Mn accumulation, and region directly basal to the symptomatic region, respectively. Concentrations of total leaf section used for analysis were also measured in control and diseased leaf samples. Every dot point represents an independent leaf section for each time point, with standard error plotted over the dot points. See Supplementary Fig. S5 for indicated ROIs on an XFM three-colour image.

Following from our demonstration that XFM elemental mapping can be used to assess nutrient distribution within and around fungi-infected leaf tissue, we sought to investigate changes in laminae nutrient distributions over the time-course of fungal infection. Not surprisingly, the changes in nutrient distribution or concentration that are observed in the fungi-induced lesion (symptomatic tissue) and the adjacent surrounding regions are not static across the 8 d time-course of infection. Below we describe the distinct patterns of variation observed in K, Ca, Mn, Fe, Cu, and Zn in the laminae which include concentrations measured in fungal mycelia, symptomatic and asymptomatic regions, and the whole sampled area for laminae from diseased and control treatments (Fig. 2; Supplementary Table S1; Supplementary Fig. S5).

In control leaf samples, there was a general trend of increasing concentration for all elements, except for K, across the 8 d time course (Fig. 2). Within the symptomatic tissue of infected leaves, changes in Ca, Fe, Cu, and Zn were mainly associated with growth and spread of the fungal mycelia, as described below. K concentration remained constant within the lesion at 2 and 4 dpi; however, a drastic decrease in K concentration was observed at 8 dpi compared with the equivalent control sample (5.1×10^4^ ng cm^–2^ versus 1.4×10^5^ ng cm^–2^), previous measurements, and concurrent measurements of the surrounding asymptomatic regions. In contrast to K, Mn concentration consistently increased within the symptomatic region as well as in the control samples at 2 dpi (389 ng cm^–2^ versus 28 ng cm^–2^) and 4 dpi (660 ng cm^–2^ versus 113 ng cm^–2^), and a further increase by 8 dpi (1307 ng cm^–2^ versus 215 ng cm^–2^) (Fig. 2; Supplementary Table S1). Indeed, the relative increase over the 8 d period was much greater for the control than for the symptomatic tissue (7.7× versus 3.4×). The difference is in the absolute change in that the symptomatic tissue had greater change than the control tissue (918 ng cm^–2^ versus 187 ng cm^–2^).

**Fig. 3.**
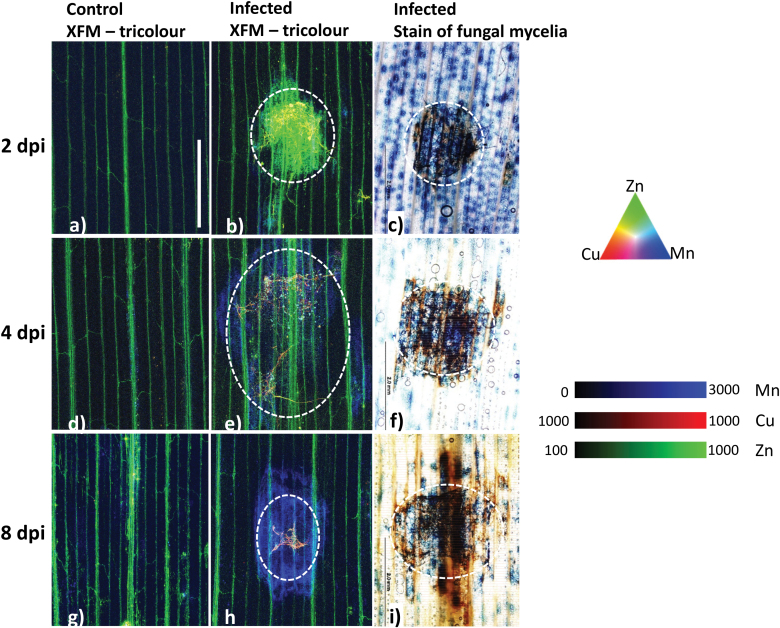
Representative three-colour overlay of XFM elemental maps for Cu (red), Zn (green), and Mn (blue) to monitor the spread of Ptr in wheat leaves over 8 d. Elemental maps for control (A, D, G) and Ptr-inoculated leaf sections (B, E, H) are shown. Representative staining of fungal mycelia within infected wheat leaf sections is also shown (C, F, I). Leaves were harvested 2 (A–C), 4 (D–F), and 8 dpi (G–I). XFM-generated Cu maps and staining of fungal mycelia in infected wheat leaf samples show that Ptr grows significantly by 2 dpi. Similarly, Mn accumulation is consistent with the browning of leaf tissue. The contrast and brightness of each elemental map are uniform in all of the representative images, with increasing and decreasing coloration or intensity depicting higher and lower concentrations of that element. To best highlight the Cu distribution in mycelia, a binary colour scale was used (i.e. 1000–1000). Scale bar=2 mm.

**Fig. 4.**
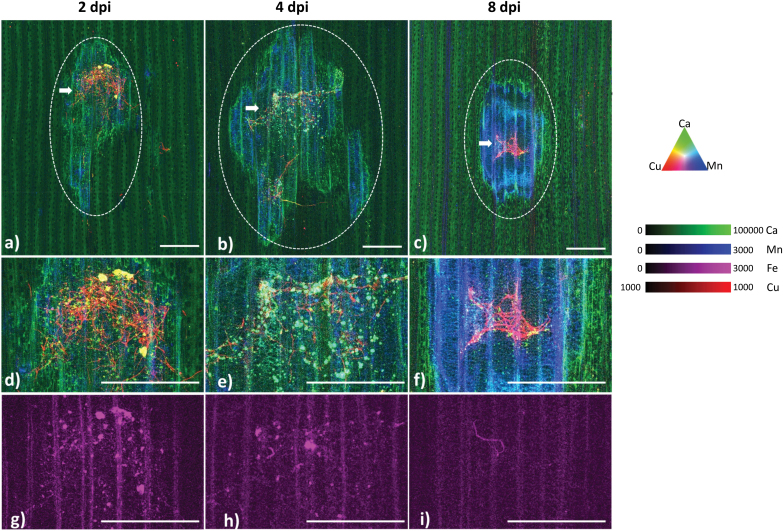
Representative three-colour overlay of XFM elemental maps for Cu (red), Ca (green), and Mn (blue) maps for Ptr-inoculated leaf sections harvested at (A) 2, (B) 4, and (C) 8 dpi. XFM-generated Ca and Fe maps show distinct microaccumulation of Ca and Fe in fungal mycelia and a general Ca accumulation in the symptomatic region in the first 4 dpi. Cu and Mn maps are included to help visualize fungal mycelia and the necrotic zone, respectively. A closer look at the region where fungal mycelia are found in (A), (B), and (C) indicated by a white arrow with a tricolour overlay of Cu (red), Ca (green), Mn (blue) in (D–F) and Fe (Magenta) in (G–I), respectively. At 2 dpi, microdeposits are evident that have Cu, Ca, and Fe accumulation. These disappear by 8 dpi as the cellular structures are no longer intact. The contrast and brightness of each elemental map are uniform in all of the representative images, with increasing and decreasing coloration or intensity depicting higher and lower concentrations of that element. To best highlight the Cu distribution in mycelia, a binary colour scale was used (i.e. 1000–1000). Scale bar=1 mm.

**Fig. 5.**
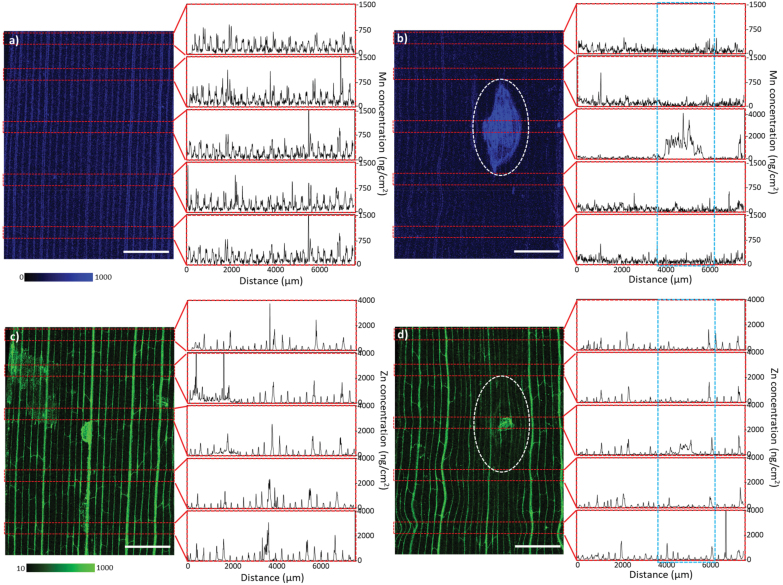
Mn and Zn elemental maps serve as a marker of physiological disruptions to a larger area beyond the lesion and presence of an impacted asymptomatic zone. XFM elemental maps for representative control and Ptr-inoculated wheat leaf samples for Mn (A, B) and Zn (C, D) harvested at 8 dpi. Concentration of Mn and Zn measured in various sections along the leaf (marked by red dashed rectangles) and provided as line plots. All plots have equivalent *x*- and *y*-axis scales except for the middle plot of Mn concentration where the accumulation of Mn reached ~4000 ng cm^–2^. In control leaf maps, Mn (A) and Zn (C) are enriched in vascular tissue, with Zn also enriched in trans-veins. The consistent structure of the vasculature is lost in the Ptr-infected leaf sample for both Mn (B) and Zn (D) maps surrounding the symptomatic zone. The enriched random spotting found on the Zn control map was due to the response of leaf to insects in the glasshouse. Scale bar=2 mm.

Large increases in Ca, Fe, Cu, Mn, and Zn concentrations were observed during the appearance of fungal mycelia within the symptomatic tissue, at 2 dpi (Figs 2–5). The rapid increase in nutrient concentration in the symptomatic tissue is attributed to fungal tissue proliferation. Interestingly, a drastic increase compared with control samples was evident in Fe (1089 ng cm^–2^ versus 160 ng cm^–2^), Cu (900 ng cm^–2^ versus 8 ng cm^–2^), and Zn (2301 ng cm^–2^ versus 114 ng cm^–2^) by 2 dpi (Figs 2–4; Supplementary Table S1). The accumulation of Ca was distinct, with spherical microdeposits that were co-localized with, or immediately adjacent to, the Fe/Cu/Zn-enriched fungal mycelia. The concentration of Ca in fungal mycelia versus the control leaf sample was 6.4×10^4^ ng cm^–2^ versus 9.1×10^3^ ng cm^–2^ at 2 dpi and 1.3×10^5^ ng cm^–2^ versus 2.0×10^4^ ng cm^–2^ at 4 dpi (Fig. 2; Supplementary Table S1). Following their initial increase, the levels of Fe, Cu, and Zn remained constant within the fungal mycelia over 4 and 8 dpi (Fig. 2), while most of the Ca and Fe microdeposits disappeared by 8 dpi (Fig. 4D–I).

The representative Cu/Zn elemental maps (Fig. 3B) and staining of fungal mycelia in infected leaf tissue (Fig. 3C) showed that the fungus proliferated considerably by 2 dpi. The size of the infected region visualized by browning of leaf tissue (Fig. 3C, F, I) and Mn accumulation (Fig. 3B, E, H) increased rapidly across the time-course.

Overall, bulk nutrient differences were detected between control and diseased leaf samples for Ca, Mn, and Zn at 2 dpi (Fig. 2). Similarly, Ca and Mn concentrations were disturbed in the asymptomatic region compared with the control leaf sample at 4 and 8 dpi (Fig. 2). In addition, highly localized and subtle changes in Mn and Zn were observed within venation of the asymptomatic tissue as described below (Fig. 3).

### XFM reveals subtle alterations in the Mn and Zn content of veins in asymptomatic leaf tissue

Besides the changes in elemental concentrations observed within the asymptomatic regions of leaf tissue described above, a wider disruption to distributions of Mn and Zn was observed in the venation apical and basal to the symptomatic region. Major alterations could be visualized within the veins of asymptomatic tissue at 8 dpi (Fig. 5). Transect profiles of Mn concentration extracted from across the width of control leaf tissue show periodic spikes in Mn concentration, consistent with or mirroring the location of the leaf vasculature (Fig. 5A). An 8-fold increase in Mn baseline concentration across the entire symptomatic zone is apparent, with a baseline shift for the periodicity of spikes aligning with the vasculature (Fig. 5B). The transects constructed in the asymptomatic tissue apical and basal to the symptomatic lesion show a reduction in the amplitude of Mn spikes (Fig. 5B). The amplitude is restored with increasing distance away from the symptomatic region (towards the leaf tip or the leaf base); however, the amplitude of the Mn spikes was lower in the tissue apical to the lesion relative to the equivalent vein basal of the lesion. This pattern was not observed in the control leaves (Fig. 5A). Contrastingly, the concentration of Mn was higher in the control leaf samples collected closer to the leaf tip compared with the base of the leaf (Supplementary Fig. S6).

Similar to Mn, major alterations in Zn distribution were also observed within veins of symptomatic and asymptomatic tissue in infected leaves. The amplitude of Zn spikes was severely hampered surrounding the infected zone compared with control leaves (Fig. 5D). A unique feature of the Zn elemental maps, in both control and infected leaves, was the ability to visualize trans-veins in the leaf (the veins that interconnect the main parallel veins). Intriguingly, the trans-veins in asymptomatic tissue contain reduced Zn compared with those of the control leaf samples, often to the extent that the trans-veins are only weakly visible, or not visible at all (Fig. 5C versus Fig. 5D). Unusual accumulation of Zn was also observed in a few of the leaf samples due to attack by leaf-feeding insects in the glasshouse. Zn distribution appears to be highly sensitive to insect attacks; however, this does not have an impact on the distribution of Zn in trans-veins (Fig. 5C).

## Discussion

It is well established that pathogenic fungi proliferate and survive in their host tissue by extracting nutrients from their hosts (Fleck *et al.*, 2011). In response, host plants have developed defence mechanisms, and one mechanism is nutrition-centric whereby the host attempts to deprive nutrient access (Cabot *et al.*, 2019) and/or actively hyperaccumulates nutrients to create a toxic environment to the fungi (Hoerger *et al*., 2013). Consistent with this, using high-resolution XFM, we observed a rapid and large re-distribution of key mineral nutrients in and around the site of infection of a common necrotrophic pathogen of wheat. Under favourable conditions for disease development (high humidity, use of a sensitive wheat cultivar, and a virulent Ptr inoculum), disease symptoms and the re-distribution of mineral nutrients were evident at 2 dpi. Indeed, for this wheat–Ptr interaction, the responses can be generalized to three distinct patterns of mineral nutrient re-distribution in and around lesions over the course of infection and disease development. Of these, the asymptomatic tissue surrounding lesions displayed depletion of Ca, Mn, and Zn within 2–4 dpi. The asymptomatic region extended beyond the visible symptomatic area and would account for an increasing proportion of laminae area as disease progresses. Subsequently, it is likely that the physiological changes within this region are important drivers of reduction in primary assimilation under disease conditions. The symptomatic zone displayed a more complex pattern of both depletion and accumulation of nutrients. In particular, Ca, Mn, Fe, and Cu showed hyperaccumulation; in contrast, large losses of K within the necrotic core of the symptomatic region were observed at 8 dpi, which probably reflects the substantial loss of cell viability and corresponding K leakage or efflux. This result is in agreement with results from studies performed with benchtop μ-XRF instrumentation that was able to map the nutrient status of soybean leaves infected with the necrotrophic fungus *Colletotrichum truncatum* (Rodrigues *et al.*, 2018). The third general pattern identified was localized (hyper-)accumulation or deposits in tissue immediately adjacent to fungal mycelia or within the mycelia by 2 dpi.

Within the symptomatic region there were two clear patterns of Ca accumulation. The first pattern was elevated accumulation at the periphery of the lesion probably associated with ROS bursts and cell death, a strategy used by necrotrophs to successfully colonize their host (Huysmans *et al.*, 2017; Li *et al.*, 2018). Chloroplasts are also involved in generating bursts of ROS for localized cell death (Zurbriggen *et al.*, 2009) and, in the case of a necrotrophic pathogen, Ptr effectors have been shown to directly disrupt light reactions in the chloroplast, resulting in ROS generation (Manning *et al.*, 2009). This may explain the accumulation of Ca surrounding the fungal mycelia with more pronounced accumulation at the periphery of the symptomatic region (Fig. 4A–C).

The second pattern of Ca distribution observed in the form of microaccumulations aligned well with the Cu-enriched fungal mycelia (Fig. 4D, E). The fungus *Sclerotinia sclerotiorum* produces oxalic acid as an invasion strategy, contributing to the formation of calcium oxalate crystals that are evident in infected stems of *Brassica carinata* in very early stages of infection (Uloth *et al.*, 2015). Similarly, Ca-oxalate crystals were visualized during plant infection using XFM (van der Ent *et al.*, 2018). We speculate that the micro-Ca accumulations observed immediately adjacent to fungal mycelia (Supplementary Fig. S7) in this study are likely to be Ca microcalcifications and worthy of confirmation in a future study.

Although the size of necrotic lesion increased over the 8 d, interestingly, Cu and Zn accumulations in the fungal mycelia did not show a change between 2 and 8 dpi. In a previous report, accumulation of Zn and Cu was shown in mycorrhizal fungi and in root inner cortex, evidence of where the proliferation of the fungus was greatest (Yun *et al.*, 1998), which mirrors the results of our study. The authors also suggested the use of Zn and Cu as surrogate measures to detect the presence of fungi, which in our case is also true for identifying Ptr mycelia in infected wheat leaf.

The origin of the Cu and Zn accumulations in the fungi in this study is not known with certainty, but it may be due to Cu–Zn superoxide dismutase (SOD). Cu–Zn SOD is involved in inactivating superoxide anions (Xu and Chen, 2013). The genome of the pathogenic fungus *Fusarium graminearum* is known to contain five SOD genes, and the cytoplasmic Cu–Zn superoxide dismutase (SOD1) has been deemed essential to fungal growth and successful infection of wheat heads (Yao *et al.*, 2016). For *F. graminearum*, Yao *et al.* (2016) suggested that SOD1 was essential in detoxifying the intracellular superoxide radicals and enabling production of deoxynivalenol which was required for virulence of the pathogen. The Cu–Zn SOD is also shown to be an essential virulence factor in *S. sclerotiorum*, and its mutation resulted in sensitivity to metals and oxidative stress (Xu and Chen, 2013). A similar strategy may be employed by Ptr given that the concentrations of Cu and Zn were relatively similar in the fungal mycelia.

The reasons for the cessation of Cu and Zn (and Fe) accumulation in fungi across 4 and 8 dpi are not known. However, one possible explanation is a restriction of nutrient availability, such as Zn supply. Zn content was observed to decrease in the trans-veins in the asymptomatic tissue, which may be a host mechanism to reduce the ability of the fungus to access plant Zn stores or fungi driven. Although only speculation at this stage, these results clearly highlight the ability of XFM to visualize alterations in Zn homeostasis between the fungus and plant tissue.

Finally, the changes in Mn distribution throughout the time-course in this study were also striking. The concentration of Mn continued to increase in the symptomatic region over 8 d. At this stage, we can only speculate on the underlying cause of these alterations; however, a pathogen-driven origin for the Mn accumulation seems likely. Mn is essential for plant tissue at trace levels, but is also highly toxic. Due to this ‘double-edged sword’ effect of Mn, its distribution and concentration are tightly regulated at the subcellular level within plant tissue. Although the tight regulation is true for all micronutrients, the Mn accumulation within the symptomatic zone may therefore reflect either a breakdown in the cellular machinery regulating Mn homeostasis or, potentially, pathogen-mediated Mn accumulation. Interestingly, a large decrease in Mn signal was observed in the leaf vasculature within the asymptomatic tissue immediately apical and basal to the symptomatic lesion which accumulated Mn. In addition, even in asymptomatic tissue, at a considerable distance apical to the symptomatic lesion (towards the leaf tip), a reduced Mn concentration was still observed within the veins. Although the specific mechanism driving the Mn accumulation in the symptomatic tissue remains unknown, these findings strongly suggest the tissue vasculature as the supplier of the Mn in wheat leaves. Further, due to the essential nature of Mn for healthy plant growth, our results indicate that as a consequence of Mn accumulation within the symptomatic lesion, Mn deficiency can propagate further along the leaf, possibly with ongoing consequences for plant health. These results clearly highlight an important role for XFM in future studies to investigate if wheat genotypes containing different propensities for Mn transport may afford additional resistance or susceptibility to fungal infection.

We have demonstrated the utility of XFM to generate high spatial resolution mapping of macro- and micronutrients in a time-course fungal infection in wheat leaves. Overall, our results show that the changes in nutrient distribution between plant and pathogen as well as the symptomatic and asymptomatic leaf tissue are dynamic, and major re-distributions of nutrients occur by 4 dpi. There is clear evidence of the importance of nutritional immunity in suppressing pathogen attacks based on use of bulk tissue-extracted nutrients (Gerwien *et al.*, 2018; D’Attoma *et al.*, 2019). However, tools that provide the ability to visualize changes in nutrients at the cellular level *in situ* in plant and fungal tissues simultaneously are highly advantageous to study competition for nutrients between plant and pathogen during pathogenesis. To our knowledge, this is the first detailed report of high-resolution mapping of mineral nutrients in fungal foliar disease.

### Conclusions and future directions

This work demonstrates the versatility of synchrotron XFM as a non-invasive tool for high-resolution mapping of essential nutrients in diseased wheat leaves. The tool enables spatial and temporal monitoring of nutrient re-distribution during plant–pathogen interactions. Using a necrotrophic foliar fungal disease of wheat as a case study, we were able to generate XFM elemental maps to monitor nutrient acquisition and distribution dynamics of the host and pathogen. The methodology and observations described here allow *in situ* analysis of mineral nutrients with minimal sample preparation, and open up the opportunity to study the spatiotemporal distribution of nutrients between plants and pathogens. In this case study, we have shown the progress of disease dynamics in a susceptible host. In future studies, a minimum of three biological replicates, treatment of one consistent region of leaf per replicate, incorporation of genetically diverse hosts, hormone signalling, and gene expression analyses will enable in-depth study of cellular dynamics that potentially result in nutritional immunity. Furthermore, the addition of genetically diverse hosts as well as other pathosystems will form the basis of cross-comparison between disease models to identify common traits that result in disease tolerance.

## Supplementary data

The following supplementary data are available at *JXB* online.

Table S1. Concentration of nutrients (ng cm^–2^) measured using XFM elemental maps for various regions of interest as indicated in Supplementary Fig. 5.

Fig. S1. Leaf samples from the control and infected treatments were mounted onto the single aperture Perspex mount with 100 mm×100 mm aperture area using double-sided tape.

Fig. S2. A screen caption of a typical X-ray emission spectrum from the wheat samples (*x*-axis is energy in keV, *y*-axis is X-ray counts).

Fig. S3. Signal depth for the elements. 

Fig. S4. A comparison of transition metal elemental maps expressed in areal density compared with elemental maps normalized to X-ray scatter, to account for variations in sample thickness

Fig. S5. XFM three-colour image with highlighted regions of interest (ROI) used for measuring the concentration of a given element.

Fig. S6. Comparison of the concentration of Mn in the total leaf area scanned in control and diseased samples.

Fig. S7. A microscope-generated image of a crystal intertwined with fungal mycelia in wheat leaf sample infected with Ptr, 4 dpi.

erab005_suppl_Supplementary_Figures_S1-S7

erab005_suppl_Supplementary_Table_S1

## Data Availability

Raw data files are available from the corresponding author upon request.
