# Supplementary material for: Synchrotron X-ray fluorescence microscopy-enabled elemental mapping illuminates the ‘battle for nutrients’ between plant and pathogen
Source: J Exp Bot. 2021 Jan 13;72(7):2757–68. doi: 10.1093/jxb/erab005 (PMC8006550; doi:10.1093/jxb/erab005)
Supplement: erab005_suppl_Supplementary_Figures_S1-S7 [file erab005_suppl_supplementary_figures_s1-s7.pdf]

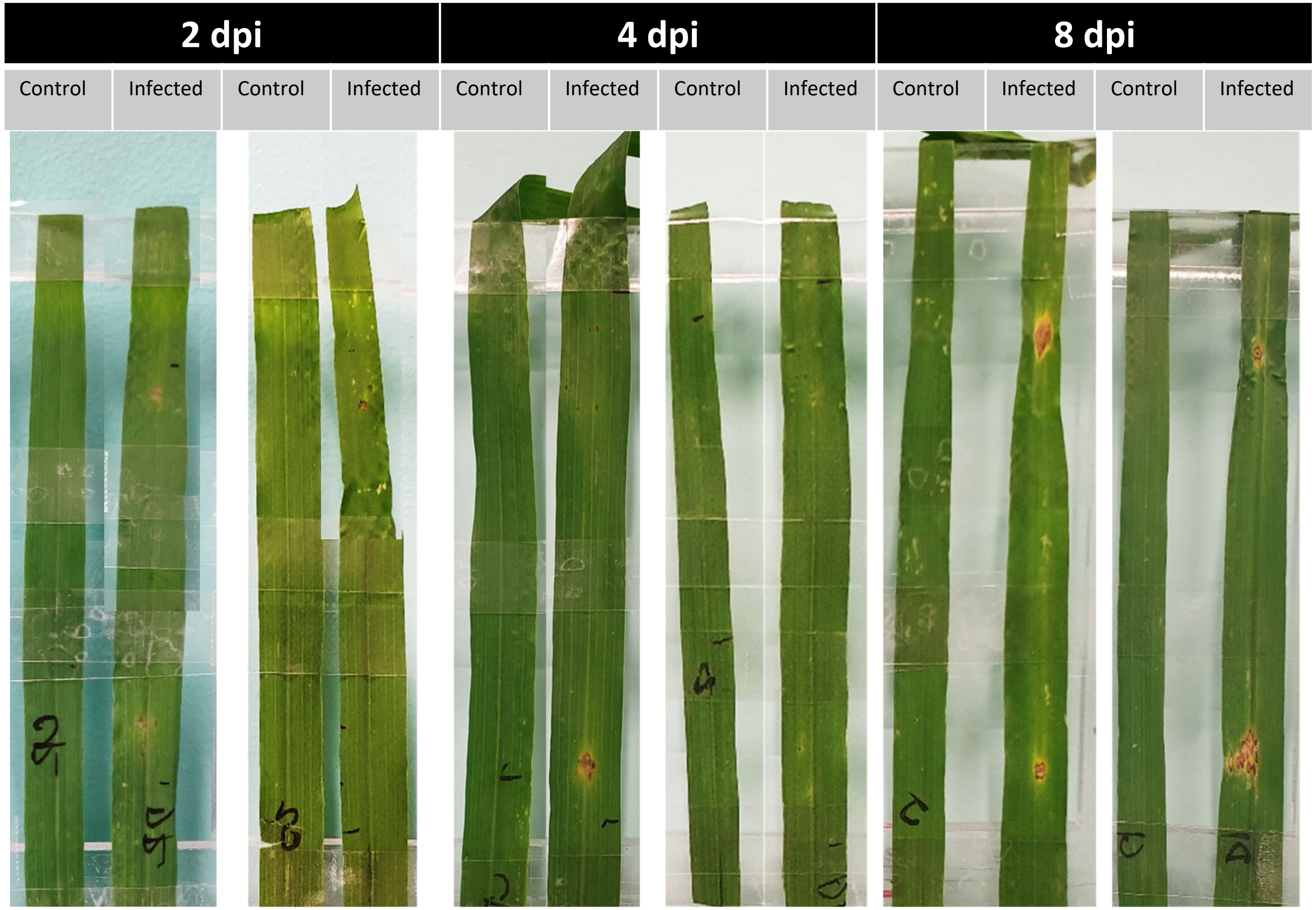

Supplementary Figure 1. Leaf samples from the control and infected treatments were mounted onto the single aperture Perspex mount with 100 mm x 100 mm aperture area using double sided tape.

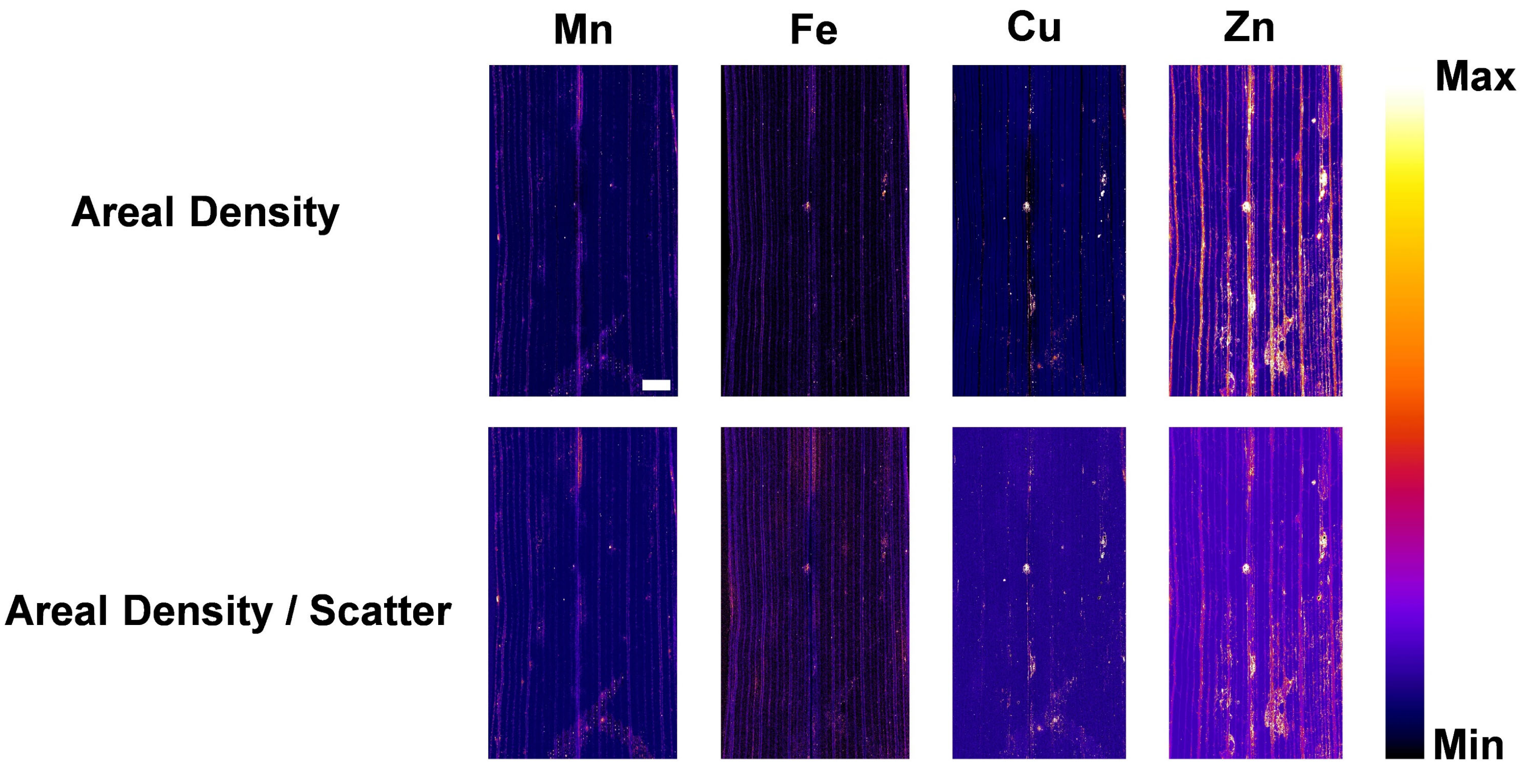

Supplementary Figure 2. A screen caption of a typical X-ray emission spectrum from the wheat samples (X-axis is energy in keV, Y-axis is X-ray counts). The count rate was kept at < 10k counts per second, per individual detector-pixel on the Maia detector. An enlarged view of the white box reveals the results of GeoPIXE elemental fit (green trace = raw data, red trace = fit). Full details of the GeoPIXE fitting process are described in Ryan 2000. Scale bar = 500 um.

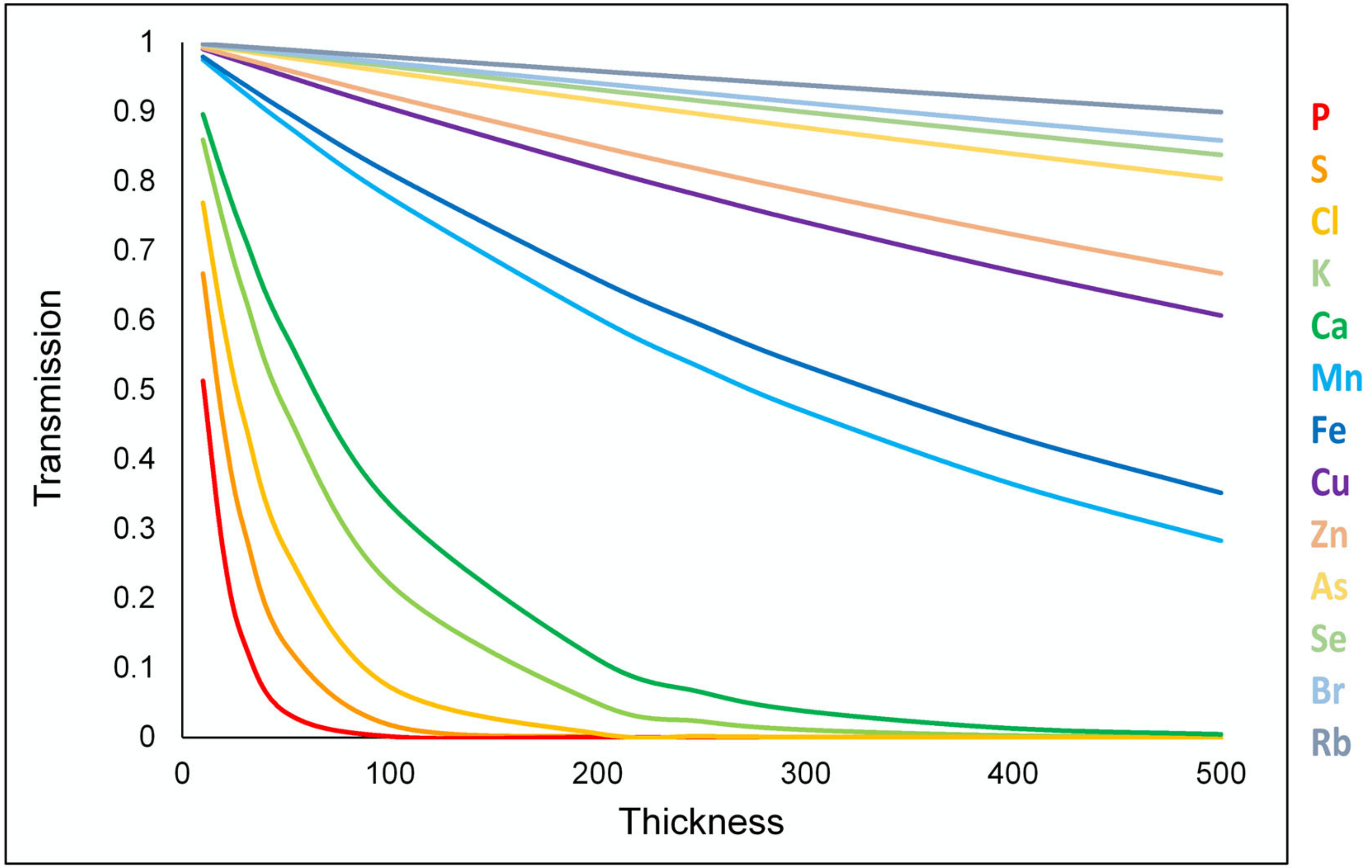

Supplementary Figure 3. Signal depth for the elements. Transmission plot for single elements (P, S, Cl, K, Ca, Mn, Fe, Cu, Zn, As, Se, Br and Rb) at given leaf thickness in order of increasing Kα. Transmission of K and Ca are 47% and 58% respectively for 50 µm thickness compared to 4.9% and 11% at 200 µm thickness. Heavier metals (Mn, Fe, Cu, Zn, As, Se, Br and Rb) have >88% and > 60% at 50 µm and 200 µm leaf thickness, respectively.

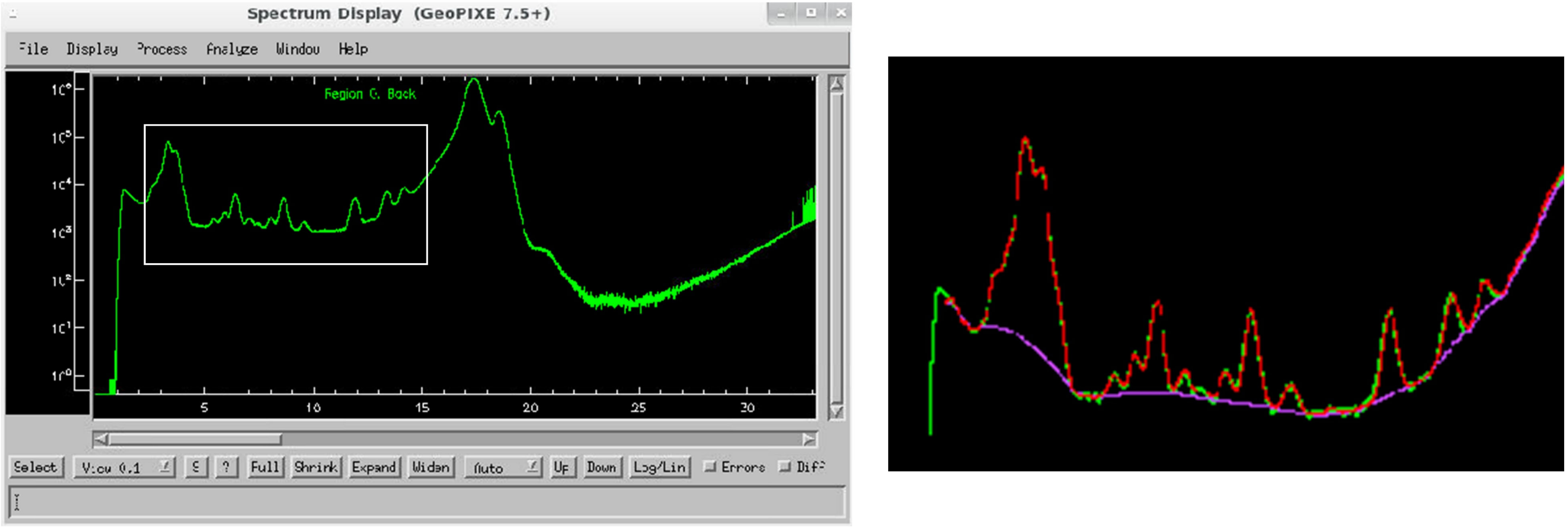

Supplementary Figure 4. A comparison of transition metal elemental maps expressed in areal density compared to elemental maps normalised to X-ray scatter, to account for variations in sample thickness. In general, regions of elemental enrichment are the same between both datasets, indicated that tissue thickness is not the source of regional variation in elemental content. Scale bar = 500 um.

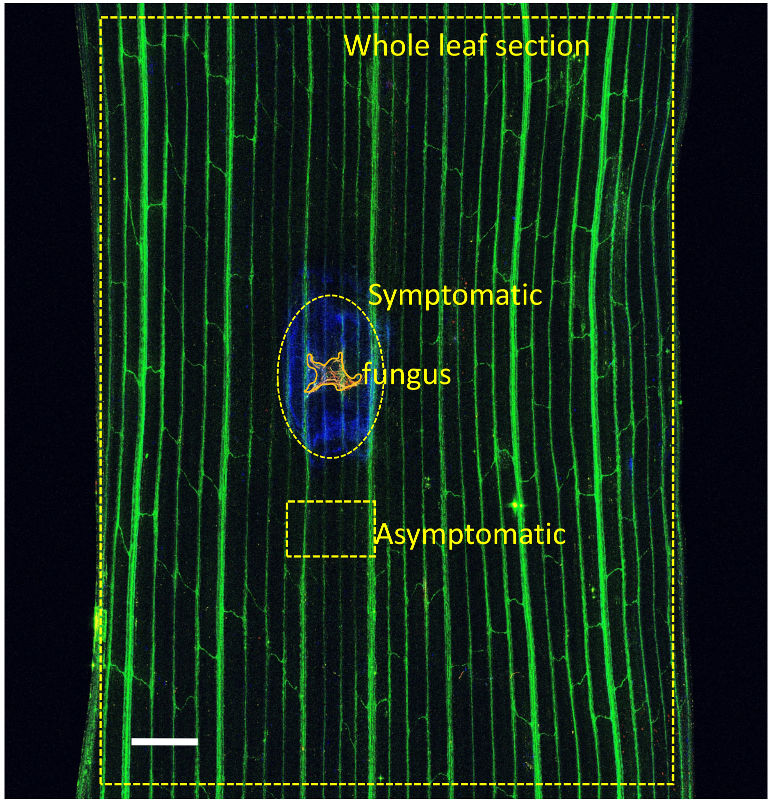

Supplementary Figure 5. XFM three-colour image with highlighted regions of interest (ROI) used for measuring the concentration of a given element. Scale bar = 1 mm.

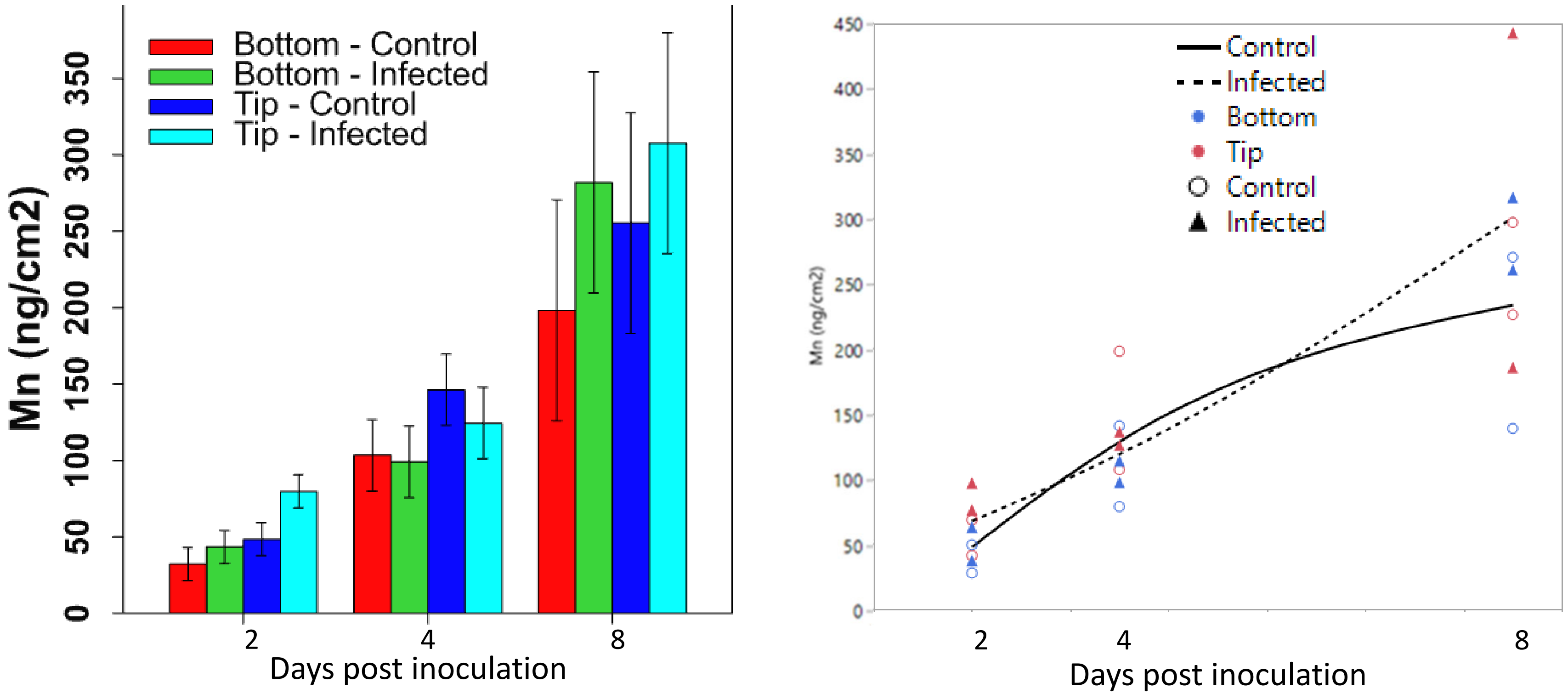

Source	LogWorth	Pvalue
Days post inoculation	7.208	0.000
Region of leaf	1.427	0.037
Treatment	0.761	0.173
Plant	0.230	0.589

Supplementary Figure 6. Comparison of the concentration of Mn in the total leaf area scanned in control and diseased samples. The two regions of wheat leaf blade scanned are referred to as the bottom (closest to the auricles) and the tip (closest to the tip end of the leaf).

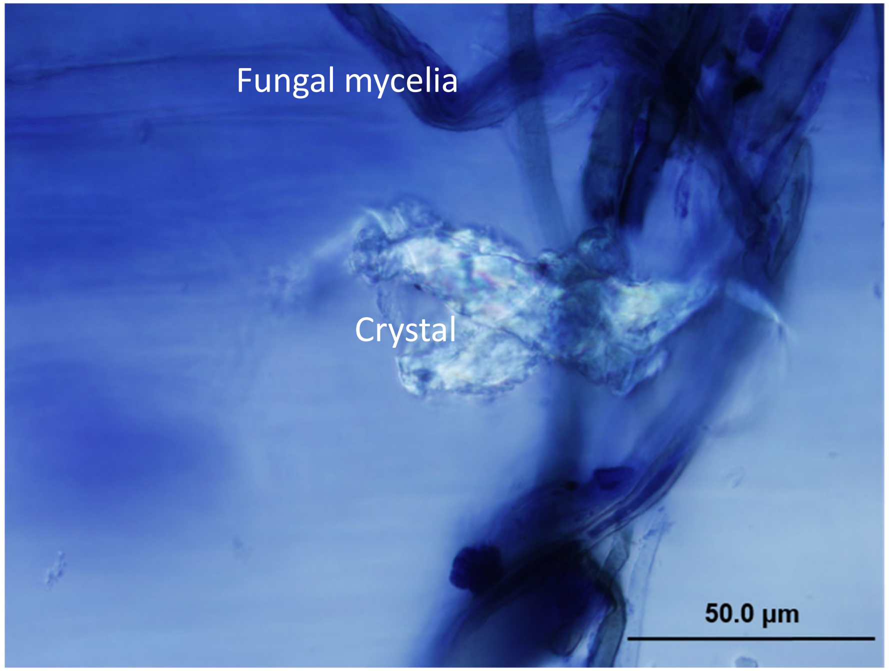

Supplementary Figure 7. A microscope image of a crystal intertwined with fungal mycelia in wheat leaf sample infected with Ptr, 4 dpi. Fungal mycelia stained with trypan blue and crystal visualised using polarising light settings of Olympus BX51 microscope.
